# Longitudinal Change of PTSD Symptoms in Community Members after the World Trade Center Destruction

**DOI:** 10.3390/ijerph16071215

**Published:** 2019-04-04

**Authors:** Rebecca Rosen, Zhaoyin Zhu, Yongzhao Shao, Mengling Liu, Jia Bao, Nomi Levy-Carrick, Joan Reibman

**Affiliations:** 1 Department of Psychiatry, NYU School of Medicine, 550 First Ave, New York, NY 10016, USA; nlevy-carrick@bwh.harvard.edu; 2 Health and Hospitals World Trade Center Environmental Health Center, Bellevue Hospital Center, H7E, 462 First Ave, New York, NY 10016, USA; zhaoyin.zhu@nyu.edu (Z.Z.); yongzhao.shao@nyulangone.org (Y.S.); Mengling.Liu@nyulangone.org (M.L.); jia.bao@nyulangone.org (J.B.); joan.reibman@nyumc.org (J.R.); 3 Department of Population Health, NYU School of Medicine, 180 Madison Ave., New York, NY 10016, USA; 4 Department of Environmental Medicine, NYU School of Medicine, 550 First Ave, New York, NY 10016, USA; 5 Department of Medicine, NYU School of Medicine, 550 First Ave, New York, NY 10016, USA

**Keywords:** PTSD symptom change, PCL score, longitudinal analysis, PTSD cluster, WTC survivors, 9/11 disaster

## Abstract

The World Trade Center (WTC) Environmental Health Center (EHC) is a treatment program for community members with exposure to the 9/11 terrorist attack and its physical and emotional aftermath. Compared to the general responders program, the WTC EHC is diverse with equal gender distribution, representation of many races and ethnicities, and a wide range of social economic status. Patients in the WTC EHC were initially enrolled for physical symptoms, most of which were respiratory, however a large portion of the enrollees scored positive for probable posttraumatic stress disorder (PTSD). In this paper we identify patient characteristics associated with probable PTSD. We also determine the characteristics associated with the longitudinal change of PTSD symptoms, including persistence and remittance, using the widely used Posttraumatic Check List-17 (PCL) cut-off value of 44, as well as changes in PCL total score and symptom cluster scores in patients of Low and High PTSD symptom severity. Few patients with elevated scores achieved a score below 44. However, longitudinal improvement in PCL score at follow-up was identified for patients with High PTSD scores (PCL > 57.5). Changes in PCL symptom clusters differed between those with High and Low PCL scores. These data suggest improvement over time in PCL score that differs depending on the severity of the score and variable responses in the PCL symptom clusters.

## 1. Introduction

The terrorist attack on the World Trade Center (WTC) on 11 September 2001 resulted in a vast environmental disaster with the collapse of the WTC towers. Local community members and first responders had potential for both acute massive dust inhalation from the dust clouds created as the WTC buildings collapsed (WTC dust cloud), as well as chronic inhalation and topical exposure from re-suspended dust and fumes from the fires that burned for months [1,2,3,4]. Exposed community members include local residents, local workers, school children, and those passing by as tourists or commuters. Well-described adverse medical health effects in this population include persistent lower respiratory symptoms (LRS) [5,6,7,8,9,10,11]. Many of these community members witnessed destruction, death and dismemberment and often experienced fear of their own death as they escaped collapsing buildings or engulfment by blinding dust clouds. Some individuals were exposed to extended rescue and recovery efforts, or displacement from homes and workplaces due to clean up efforts of damaged or contaminated buildings. Well-described mental health symptoms in the community include those associated with posttraumatic stress disorder (PTSD), depression, and anxiety [6,12,13]. 

The World Trade Center Environmental Health Center (WTC EHC) was established to treat and monitor the health conditions of community members that resulted from exposure to the event and its aftermath [14]. The initial funding of the program limited inclusion in the treatment program to those with physical symptoms [4,11]. As a result, patients with exposure-based mental health symptoms but no physical symptoms were not eligible for enrollment in the treatment program during its initial years. Despite this, from the program’s beginning, the Initial and Monitoring visit evaluations included assessment of both physical and mental health symptoms. 

We have previously described mental health symptoms in the WTC EHC population [13]. We now report the longitudinal change of PTSD symptoms in our original cohort who self referred for physical complaints. Our goal was to identify factors associated with persistent or remitted PTSD symptoms over two time points including demographic characteristics, WTC exposures and baseline severity of a PTSD screening instrument score. We also examined differences among changes in symptom clusters consistent with PTSD heterogeneity. 

## 2. Methods 

### 2.1. Subjects

Patients who enrolled in the WTC EHC at Bellevue Hospital between August 2005 and February 2009 were included in the analysis. At that time, a physical complaint was the only criterion for enrollment [4,11]. The Institutional Review Board of New York University School of Medicine approved the research database (NCT00404898) and only data from patients who signed informed consent were used for analysis. Patients were included in the analysis if they had data including WTC exposure, physical and mental health questionnaires from an Initial visit between August 2005 and February 2009, and a subsequent Monitoring visit. All Monitoring visits were between October 2009 and May 2016.

### 2.2. WTC Exposures and Medical Assessment

Upon enrollment in the WTC EHC, patients completed a multi-dimensional, interviewer-administered questionnaire that included demographic information and characterizations of WTC-related exposure [11]. Individuals were classified as positive for dust cloud exposure if they reported having been in a WTC dust cloud created by the collapsing buildings on 11 September 2001. Potential for WTC acute and chronic exposures were characterized by classification into four additional categories: local resident (resident), local worker, cleanup worker and other. 

Patients who reported more than 5 pack-year history of tobacco use were defined as ever smokers. Body mass index (BMI) of patients was calculated using information gathered during the Initial medical visit. The presence and severity of lower respiratory symptoms (LRS) of wheeze, cough, chest tightness and dyspnea, as well as the upper respiratory symptom of rhinitis or sinusitis were measured by standardized health questionnaires [11]. Patients with symptoms more than twice per week during the month preceding enrollment were considered positive for any LRS or sinus symptoms. Spirometry was performed in accordance with American Thoracic Society/European Respiratory Society standards [15] on a Viasys Vmax spirometer (Yorba Linda, CA). Patients were categorized as abnormal if they had a reduced forced vital capacity (FVC), reduced forced expiratory volume in one second (FEV_1_), or a reduced ratio of FEV_1_/FVC [16].

### 2.3. Mental Health Symptom and Treatment Assessments

The Posttraumatic Check List-17 (PCL) [17] was used to measure PTSD symptom presence and severity. A score ≥ 44 was considered positive for probable PTSD (PTSD+), and a score < 44 was considered negative for probable PTSD (PTSD−) [9]. We evaluated the longitudinal change in PCL score using a comparison of PCL scores at Initial and Monitoring visits. We defined those who scored positive for probable PTSD at both Initial and Monitoring visits as Persistent PTSD and those who scored positive at their Initial visit and negative at their Monitoring visit as Remitted PTSD. PTSD symptom severity was further defined by the level of the initial PCL score as follows: < 44, No PTSD; 44–57.5, Low PTSD; > 57.5, High PTSD. The “Low” and “High” categories were defined using 57.5 as the cut point as this number is the median score among those with a score ≥ 44. Questions from the PCL-17 were also matched to the DSM-V diagnostic criteria for characterization into four clusters as previously described [13], reflecting symptoms of re-experiencing, avoidance, negative cognitions/mood, and arousal. An average score for each patient was calculated for each cluster, ranging between 1–5. 

We used the Hopkins Symptom Checklist for depression and anxiety (HSCL-25) [18]. These scales provided scores of depression (HSCL-D) and anxiety (HSCL-A) severity with a score ≥ 1.75 considered to suggest probable depression or probable anxiety. The CAGE Questionnaire was used to screen for problem alcohol use, with a score ≥ 2 used to categorize persons with probable alcohol abuse [19].

We collected self-reported mental health treatment information during the Monitoring examination. Treatment for a mental health condition was defined to include any type of reported treatment. Treatment was included if it was reported as individual psychotherapy for ≥ one month, group psychotherapy treatment for ≥ 2 months, or prescribed pharmacologic treatment. 

### 2.4. Statistical Methods

Categorical variables were summarized using counts and percentages and the significance of between-group difference was assessed by Chi-square test. Continuous variables were summarized using median and interquartile range (IQR) and difference between independent groups was assessed by Wilcoxon rank-sum test (i.e., Mann-Whitney test), and between correlated groups using Wilcoxon signed rank test. Logistic regression was used to quantify the association between binary outcomes (e.g., probable PTSD) and covariates of interest. Linear regression was used to quantify the association between continuous outcome (e.g., PCL score) and covariates of interest. Multivariate models were constructed using selected covariates that were significant in univariate analyses. Observed mental health treatment was not randomized in this study cohort and therefore direct comparison of treated versus untreated groups would not be meaningful due to potential associated biases. To overcome this issue, we used the propensity score method to discuss potential existence of treatment effect on the longitudinal changes of PCL scores. A value of P < 0.05 was used to test for two-sided statistical significance. All statistical analyses were conducted using SAS, version 9.4 (SAS Institute, Cary, North Carolina, United States). 

## 3. Results

### 3.1. Patient Characteristics

WTC exposure history, physical and mental health data gathered at Initial and Monitoring visits were available for 738 patients. The median time between visits was 3.43 years (Q1, Q3; 2.88, 4.32). Most patients (Table 1) were > age 45 (69%), with a similar number of males (49%) and females (51%). Many were Hispanic (44%), had an annual individual income of < $30,000 and had a ≤ 12th grade level of education (40%). Local workers comprised the largest exposure category (47%). Fifty per cent reported having been caught in the WTC dust cloud. Most patients (90%) had LRS, and 57% reported sinus symptoms. Lung function was within normal limits in most (64%). 

As noted above, the criterion for enrollment in this early program was the presence of physical, but not mental health symptoms. Despite this, many patients scored positive for probable PTSD (PTSD+; 38%, *n* = 284), depression (55%), and anxiety (38%) at their Initial visit. Symptoms suggestive of alcohol misuse were reported in 7% of the population. Categories were not mutually exclusive.

### 3.2. Characteristics Associated with PTSD Symptoms at Initial Visit 

We compared demographic characteristics, medical symptoms, lung function and comorbid conditions in PTSD+ (*n* = 284) and PTSD− (*n* = 454) patients at their Initial visit (Table 1; see methods section for category description). Patients who self-reported as Hispanic (*p* = 0.001), had income ≤ $15,000/year or ≤ 12th grade education were more likely to score PTSD+ (*p* < 0.001, 0.003 respectively). The presence of any LRS symptom (*p* = 0.001) was associated with being PTSD+. Those PTSD+ were also more likely to report probable depression and anxiety (both *p* < 0.0001) and to report alcohol misuse (*p* = 0.02). In a multivariate logistical regression using any significant variable, the presence of physical symptoms of LRS (OR = 2.21; *p* = 0.05), as well as co-morbid mental health symptoms of probable depression (OR = 18.11; *p* < 0.0001), probable anxiety (OR = 4.64; *p* < 0.001), or alcohol misuse (OR = 2.45; *p* = 0.05), remained significant (data not shown).

### 3.3. Demographic and WTC Exposure Characteristics Associated with Persistent and Remitted PTSD

As shown (Table 2), 279 patients who scored PTSD+ at their initial visit could be classified as Persistent or Remitted as described in the methods section (5 patients with incomplete baseline characteristics were excluded from analysis). The majority of patients (*n* = 214) remained Persistent, while few patients (*n* = 65) fit criteria for Remitted. Race/ethnicity and baseline depression status were associated with being Persistent (*p* = 0.01 and 0.04 respectively) in univariate analysis. No covariates were significant in multivariate analysis (data not shown).

### 3.4. Longitudinal Assessment of Severity of PTSD Symptoms 

A dichotomous evaluation as Remitted or Persistent may be insensitive to a heterogeneous decrease of the PCL score. We therefore directly studied changes in the PCL score. Patients were grouped into severity categories based on their PCL score at Initial visit as No PTSD, Low PTSD, or High PTSD (Table 3; see methods section for description of grouping). High PTSD patients had a 9-point reduction in the median score between visits (*p* < 0.0001), consistent with a statistically significant improvement in PCL score. In contrast, the decrease in median PCL score in the Low PTSD patients did not reach significance. Despite these improvements, the median score at Monitoring remained ≥ 44 in both groups. There was a small but statistically significant increase of median PCL score among the No PTSD group, and the median score at Monitoring remained < 44. 

The possibility exists that treatment modified the PCL score over time. Our study was not designed to evaluate treatment, however we used the observed treatment status, as defined in the methods section, to develop propensity scores from logistic regression with relevant predictors including race/ethnicity, income, and initial mental health scores included in the model. High propensity scores of being treated were positively correlated with decreases in longitudinal PCL scores (*p* < 0.0001; data not shown). 

### 3.5. PTSD Symptom Clusters Over Time

The heterogeneity within PTSD can also be described by the symptom clusters that comprise the overall score. We therefore examined change in score of each PTSD symptom cluster within our PTSD severity categories (Table 4). Patients with Low PTSD showed significant improvement only in the arousal cluster score (*p* = 0.01); we were unable to detect a significant change in the score of any of the other clusters. In contrast, those with High PTSD improved significantly on all clusters (re-experiencing, *p* = 0.0003; avoidance, *p* = 0.001; negative thoughts/emotions and arousal, *p* < 0.0001). A slight increase in all cluster scores was noted in those who scored below threshold on initial visit.

## 4. Discussion

In this paper we characterize mental health scores in patients who had enrolled in a community program for physical symptoms after a traumatic event and describe characteristics associated with PTSD symptom scores in this population, as well as changes in scores over time. Our population was a diverse race/ethnic population with many of low income and education. Importantly, despite enrollment in the program for physical symptoms, many patients scored positive for probable PTSD. We did not observe many patients with remittance of PTSD symptoms defined as a score below 44, however we identified patients who had improvement in PCL scores, particularly in those who were more symptomatic as reflected in higher PCL scores on enrollment in the program. Moreover, we showed improvement in each PTSD symptom cluster score in patients with more severe PTSD symptoms, whereas those with milder PTSD symptoms had more limited improvement. 

We identified many patients who scored positive for probable PTSD despite enrolling in a program for physical symptoms. High rates of PTSD have been well described in civilian populations who are not trained for trauma [9,20,21,22]. Our patients were recruited for physical symptoms and were not specifically seeking mental health treatment. High mental health scores were only incidentally reported, reinforcing the need to perform mental health screening in all disaster-exposed populations. Moreover, these high rates may in part be due to the diversity of the population, including many with low income and education, factors previously reported to be associated with the development of PTSD [21,23]. In addition, the presence of co-morbid physical symptoms, including lower respiratory symptoms, and mental health symptoms was also associated with probable PTSD, reinforcing the need to consider co-morbid presentations [6,24,25]. 

Few studies have examined the longitudinal change in mental health symptoms in WTC community members, [6,26,27,28,29] particularly in those who have sought care for physical, or even mental health symptoms as a result of their exposure. To begin to analyze the change in symptoms over time in our highly affected population we used a simple cut-off value that is often used to determine whether an individual should undergo further evaluation, and has been used in numerous epidemiologic studies in WTC-exposed populations [9]. Most (77%) of the patients in the study remained symptomatic when the group was dichotomized into Persistent or Remitted based on this score. This finding suggests that these symptoms were refractory as has been described in some WTC exposed populations [22,30] and underscores the importance of studying and improving patient management. Alternatively, this finding may suggest that this dichotomous evaluation is insensitive for the identification of more subtle changes in the level of PTSD symptoms. Indeed, we noted changes in PCL scores when evaluated as a continuous variable in subgroups of patients. In addition, the PCL may have low sensitivity in identifying functional improvement. 

There was wide variation in the PCL score in our population, suggesting heterogeneity within the severity of symptoms. When we compared the change in PCL score as a continuous variable, patients who had a higher score at their Initial visit had a significant improvement in their score. This finding was not seen in those who began with a lower score. Importantly, neither group reduced their median score below our cut-off threshold. This finding suggests that there is improvement, although there may to be differences in responsivity. 

Our study was not designed to evaluate treatment, however, we used the observed treatment status to develop propensity scores from logistic regression with relevant predictors and showed that high propensity scores of having received treatment were positively correlated with decreases in longitudinal PCL scores. These data suggest a relationship between treatment and improvement in PCL score and reinforce the need for clinical trials to study the role of individual treatment modalities and potential treatment effect. 

We have previously suggested heterogeneity of PTSD within this community population [13]. However, few studies examine longitudinal changes in the individual components that comprise PTSD. Symptom cluster trajectories have been shown to have a variable course in, for example, traumatically injured populations [31]. We show an improvement in score in those with High PTSD at initial evaluation, suggesting that there may be differences in the components of these higher scores that can be modified. When we evaluated this question by examining changes within symptom clusters, patients with higher PCL scores at Initial visit showed improvement across all clusters. This finding suggests that patients with greater PTSD symptom severity at Initial visit have responsiveness across all components of PTSD. Patients with lower PCL scores reflecting less symptom severity only improved significantly on the arousal cluster, suggesting that this cluster of symptoms may more easily attenuated. Their failure to completely return to levels below threshold suggest the possibility of some level of intractability.

There are limitations to this study. We studied a civilian population at only two time points, limiting the ability to identify fluctuations in symptom presentation. The population under study was exposed to the same traumatic event, which is a strength of the study. However, this unique exposure may also limit the generalizability of these results. Participants were self-referred patients seeking treatment for physical symptoms related to exposure to the WTC disaster and were not selected from a wider group of WTC exposed community members, raising the potential for selection bias for these symptoms. The possibility exists, too, that the role of treatment was inadequately evaluated and patients with higher scores might have been more likely to have received treatment, with subsequent bias, in our evaluation. We used the PCL-17 to assess PTSD symptoms. Although this is a commonly used tool, it can be subject to recall bias, sensitivity, and specificity [32]. Certain variables were not completely assessed and included in the analysis, such as pre and post-trauma variables. These limitations reinforce the need for more studies. 

## 5. Conclusions

Our goal was to understand longitudinal patterns of PTSD in a cohort of WTC exposed community members seeking help for physical ailments related to the exposure. Many patients scored positive for probable PTSD, which was associated with physical and mental health comorbidities. Few patients remitted, when analyzed as a threshold score, and no we could not identify variables associated with remittance or persistence after multivariate analysis. However, we detected improvement in scores in patients with persistence; the decrease was significant for those with more severe PTSD symptoms. Heterogeneity in response was also observed in changes in PTSD symptom clusters, where we showed improvement in each PTSD symptom cluster score for patients with more severe PTSD symptoms, whereas those with milder PTSD symptoms improved only on the arousal cluster. 

An understanding of the patterns of longitudinal PTSD symptoms is important for trauma management in this and other populations. Identifying the variability in changes in PTSD symptom clusters can also help to target treatment. Moreover, studies suggest that PTSD symptoms do not remain stable over a lifetime, but may change and even reactivate with aging, suggesting the need for further study evaluation [33]. Understanding the patterns of longitudinal symptoms in this cohort will assist in identifying patients at high risk for chronicity and in guiding timely, effective interventions.

## Figures and Tables

**Table 1:** Patient characteristics at Initial visit and comparison of PTSD+ and PTSD− patients (*n* = 738).

Demographic Characteristics	Total	PTSD+ (*n* = 284)	PTSD− (*n* = 454)	*p*-Value
Gender, *n* (%)				0.24
Female	375 (51)	152 (53.5)	223 (49)	
Male	363 (49)	132 (46.5)	231 (51)	
Age, *n* (%)				0.24
<45	229 (31)	81 (29)	148 (33)	
≥45	509 (69)	203 (71)	306 (67)	
Race/ethnicity, *n* (%)				0.001
Hispanic	323 (44)	149 (52)	174 (38)	
Non-Hispanic white	205 (28)	66 (23)	139 (31)	
Non-Hispanic black	142 (19)	45 (16)	97 (21)	
Asian	49 (7)	14 (5)	35 (8)	
Other	19 (3)	10 (4)	9 (2)	
Education, *n* (%)				0.003
≤High school	292 (40)	132 (47)	160 (35)	
>High school	445 (60)	152 (54)	293 (65)	
Income, *n* (%)				<0.0001
≤$15,000/year	301 (42)	148 (54)	153 (35)	
$15,000–$30,000/year	145 (21)	49 (18)	96 (22)	
>$30,000/year	265 (37)	76 (28)	189 (43)	
BMI, *n* (%)				0.80
≤30	466 (64)	177 (63)	289 (64)	
>30	262 (36)	102 (37)	160 (36)	
Ever smoker, *n* (%)				0.21
yes	287 (39)	118 (42)	169 (37)	
no	451 (61)	163 (58)	284 (63)	
Exposures				
WTC dust cloud exposure, *n* (%)				0.14
yes	361 (49)	148 (53)	213 (48)	
no	377 (51)	130 (47)	234 (52)	
Exposure classification, *n* (%)				0.12
Local worker	340 (47)	127 (45)	213 (47)	
Resident	140 (19)	45 (16)	95 (21)	
Clean-up worker	171 (23)	77 (27)	94 (21)	
Other	80 (11)	33(12)	47 (10)	
Symptoms				
Lower respiratory symptoms				
Any Lower Respiratory Symptoms				0.001
yes	663 (90)	266 (95)	397 (87)	
no	75 (10)	15 (5)	57 (13)	
Spirometry				0.17
normal	472 (64)	173 (61)	299 (66)	
abnormal	266 (36)	111 (39)	155 (34)	
Upper respiratory symptoms				
Sinus congestion				0.79
yes	408 (56)	153 (56)	255 (57)	
no	330 (44)	122 (44)	195 (43)	
Positive mental health score				
PTSD, *n* (%)				
yes	284 (38)			
no	454 (62)			
Depression, *n* (%)				<0.0001
yes	404 (55)	265 (93)	139 (31)	
no		19 (7)	315 (69)	
Anxiety, *n* (%)				<0.0001
yes	277 (38)	202 (71)	75 (17)	
no	461 (62)	82 (29)	379 (83)	
Cage, *n* (%)				0.02
yes	50 (7)	27 (10)	23 (5)	
no	688 (93)	250 (90)	428 (95)	

Univariate analysis. Categorical variables presented as frequencies and percentages and difference between 2 groups assessed by the Chi-Square test.

**Table 2:** Characteristics associated with Persistent and Remitted PTSD *.

Demographic Characteristics	Persistent PTSD (*n* = 214)	Remitted PTSD (*n* = 65)	*p*-Value
Gender, *n* (%)			0.20
Female	109 (51)	39 (60)	
Male	105 (49)	26 (40)	
Age, *n* (%)			0.91
<45	61 (28.5)	19 (29)	
≥45	153 (71.5)	46 (71)	
Race/ethnicity, *n* (%)			0.01
Hispanic	119 (56)	28 (43)	
Non-Hispanic white	52 (24)	12 (18)	
Non-Hispanic black	30 (14)	14 (22)	
Asian	6 (3)	8 (12)	
Other	7 (3)	3 (5)	
Education, *n* (%)			0.58
≤High school	97 (45)	32 (49)	
>High school	117 (55)	33 (51)	
Income, *n* (%)			0.16
≤$15,000/year	107 (52)	39 (63)	
$15,000 - $30,000/year	40 (19)	6 (10)	
>$30,000/year	59 (29)	17 (27)	
BMI, *n* (%)			0.25
≤30	138 (65)	36 (57)	
>30	74 (35)	27 (43)	
Ever smoker, *n* (%)			0.90
yes	89 (42)	28 (43)	
no	122 (58)	37 (57)	
Exposures			
WTC dust cloud exposure, *n* (%)			0.12
yes	117 (56)	28 (44)	
no	93 (44)	35 (56)	
Exposure classification, *n* (%)			0.37
Local worker	101 (48)	25 (38)	
Resident	32 (15)	11 (17)	
Clean-up worker	58 (27)	18 (28)	
Other	21(10)	11 (17)	
Symptoms			
Lower respiratory symptoms			
Any Lower Respiratory Symptoms			0.85
yes	200 (95)	62 (95)	
no	11 (5)	3 (5)	
Spirometry			0.30
normal	134 (63)	36 (55)	
abnormal	80 (37)	29 (45)	
Upper respiratory symptoms			
Sinus congestion			0.052
yes	123 (59)	28 (45)	
no	85 (41)	34 (55)	
Positive mental health score			
Depression, *n* (%)			0.04
yes	203 (95)	57 (88)	
no	11 (5)	8 (12)	
Anxiety, *n* (%)			0.11
yes	157 (73)	41 (63)	
no	57 (27)	24 (37)	
Cage, *n* (%)			0.94
yes	21 (10)	6 (10)	
no	189 (90)	56 (90)	

* Univariate analysis, *n* = 279 (five subjects with incomplete baseline characteristics are excluded from analysis. Categorical variables presented as frequencies and percentages and difference between 2 groups assessed by the Chi-Square test.

**Table 3:** Longitudinal assessment of PTSD score in severity categories.

PCL Status	No PTSD (*n* = 454)	Low PTSD (*n* = 142)	High PTSD (*n* = 142)
Initial (PCL1)	27 (20, 35)	51 (47, 54)	66 (62, 70)
Monitoring (PCL2)	32 (23, 41)	49 (42, 58)	57 (49, 66)
Change (PCL1-PCL2)	−4 (−12, −2)	2 (−6, 9)	9(2, 17)
*p*-value	<0.0001	0.11	<0.0001

Continuous variables presented as Median and IQR (Q1, Q3) and difference between 2 groups assessed by Wilcoxon signed rank test.

**Table 4:** PCL cluster scores at Initial and Monitoring visit in PTSD severity categories.

Symptom Severity Level	Visit	Re-Experiencing	Avoidance	NegativeThoughts/Mood	Arousal
No PTSD (*n* = 454)					
	Initial	1.4 (1.0, 2.0)	1.0 (1.0, 2.0)	1.2 (1.0, 1.8)	1.8 (1.1, 2.4)
	Monitoring	1.6 (1.2, 2.4)	2.0 (1.0, 3.0)	1.5 (1.0, 2.2)	2.0 (1.4, 2.8)
	*p*-value	<0.0001	<0.0001	<0.0001	<0.0001
Low PTSD (*n* = 142)					
	Initial	2.6 (2.2, 3.0)	3.0 (2.0, 4.0)	2.8 (2.4, 3.3)	3.6 (3.0, 3.8)
	Monitoring	2.8 (2.2, 3.4)	3.0 (2.5, 4.0)	2.7 (1.8, 3.2)	3.2 (2.6, 4.0)
	*p*-value	0.23	0.45	0.06	**0.01**
High PTSD (*n* = 142)					
	Initial	3.8 (3.4, 4.2)	4.0 (3.5, 5.0)	3.8 (3.2, 4.2)	4.2 (3.8, 4.6)
	Monitoring	3.2 (2.6, 4.0)	3.5 (3.0, 4.5)	3.1 (2.4, 3.8)	3.7 (3.0, 4.2)
	*p*-value	0.0003	0.001	<0.0001	<0.0001

Cluster scores are median of questions noted and IRQ (Q1, Q3) and difference between 2 groups assessed by Wilcoxon signed rank test.
